# The effect of mindfulness on online self-presentation, pressure, and addiction on social media

**DOI:** 10.3389/fpsyg.2022.1034495

**Published:** 2022-12-05

**Authors:** Chunhui You, Yang Liu

**Affiliations:** ^1^School of Humanities, Zhejiang University of Technology, Hangzhou, China; ^2^School of Computer, Zhejiang University of Technology, Hangzhou, China

**Keywords:** mindfulness, social network relations, self-presentation, social media pressure, social media addiction

## Abstract

As social media has become more imperative in daily life, people pay more attention to self-presentation and impression management on social media, and some have even become psychologically dependent. There is a large group of socially addicted users who continuously strive to improve their online self-presentation. Due to stress and burnout arising from social media addiction, people change their social media behavior. The influence of mindfulness on social behavior cannot be ignored. This study aims to explore coping behaviors and the role of mindfulness for people under social media pressure and social media addiction in China’s special political environment. We found significant differences in self-presentation, social media pressure, and social media addiction among different circles in the Chinese context. Experiments have shown that people’s socially addictive behaviors and abilities to withstand social media pressure are affected by their mindfulness. In addition, the more social media pressure people perceive on social media, the more likely they are to stop using social media and shift to offline interpersonal interactions. However, when there are more offline interpersonal interactions, people’s willingness to return to social media platforms increases.

## Introduction

Social media emerges to be the primary platform for people to communicate with each other. An increasing number of people use social media for social interaction, and an online network society has emerged (Castells, 2001). People use social media to shape and promote an idealized self-image. The interactive behavior of liking, sharing, and commenting replaces offline, face-to-face communication. Compared to offline communication, online social behavior is more performative. Many users share creative performances to present themselves and their talents. The content posted by users on social media gradually becomes the primary channel for others to know them and even serves as a reference for others whether they want to communicate further.

The appearance on social media might also determine whether a user can join a community (e.g., work, leisure, politics). Such communities are referred to as circles in China. The cultural, social, and political development of China differs significantly from Western countries. Therefore, the qualifications for entering a community differ. Entering a circle in China is, among other factors, driven by political affiliation and personal relations (Jiade, 2017). Political affiliation refers to membership in or close association with a political party or organization and indicates one’s political orientation and views. In comparison, personal relations are less specific than political affiliation when judging whether a person can enter a circle. Political affiliation is an objective judgment and relatively stable over time, while personal relations are affected by subjective, dynamic factors.

As social media becomes increasingly prevalent in life, many people become psychologically dependent and sometimes even addicted. They continuously strive to present themselves positively and shape an idealized self-image. Intensified social media addiction can also greatly impact offline life, because it causes stress and potentially burnout. Consequently, people develop different coping behaviors, one of which is to relieve pressure by reducing or stopping the usage of social media (Lazarus and Folkman, 1984). Some users purposively reduce the frequency of their posts or even close some of their social media accounts. Another coping behavior is to return to social media after a brief period of deactivation (Ravindran et al., 2014; Zhao, 2019).

Mindfulness describes a practice of meditation in which a person is fully aware of his or her activities and feelings in the present moment, without any subjective interpretations and judgments (Bishop et al., 2004). Recently, it becomes one of the most-researched areas in psychotherapy (Germer et al., 2013). Existing research shows that mindfulness is linked to people’s mental health (Liu et al., 2022a). According to some studies, mindfulness is associated with people’s mental health. Rasmussen and Pidgeon (2011) report that “mindfulness significantly predicts high levels of self-esteem and low levels of social anxiety.” Another study confirms that improving individual mindfulness can effectively foster subjective well-being (Liu et al., 2022b). And spirituality mediates the relationship between mindfulness and subjective well-being (Liu et al., 2020). The many benefits of mindfulness have led scholars to explore how to improve mindfulness. Research shows that mindfulness meditation can significantly increase mindfulness and boost creativity (Chen et al., 2022). In addition, studies started to explore the interplay between mindfulness and social media use. For example, research by Poon and Jiang (2020) proves that mindfulness can effectively mitigate the negative effects of social media. Previous research on mindfulness shows that mindfulness has a considerable influence on people’s social life. This study attempts to explore the interrelation between mindfulness and social media use.

Even though some studies point out the interaction between mindfulness and social media addiction (Sriwilai and Charoensukmongkol, 2016), other studies reveal a correlation between mindfulness and self-control (Bowlin and Baer, 2012). However, how mindfulness relates to the two coping behaviors described above is an open field. Previous studies have summarized the main factors that reduce people’s performative behavior on social media. For instance, the rising cost of maintaining a self-image (Hong and Duan, 2020), the increasing complexity of social relationships on social media, the hindrance of self-expression (Chen and Hu, 2015), and the complex use and operation of social media technology (Maier et al., 2015).

This study aims to discuss the following questions: 1. Are there differences in social media stress and social addiction behaviors faced by people from different political backgrounds in China’s unique political and cultural context? 2. Does individual mindfulness lead to differences in how people shape their self-image on social media? 3. How does mindfulness affect people when facing social media pressure? 4. Finally, when people become socially addicted and are thus tired of self-image construction or perceived social pressure, what behaviors do they adopt to cope with it?

## Literature review and research hypothesis

In this section, we review the related literature. We first review literature that investigates Chinese social network relations in Section 2.1 before we summarize literature on mindfulness in Section 2.2. In Section 2.3, we present literature that discusses self-presentation on social media, and Section 2.4 summarizes literature regarding social media pressure and social media addiction. Finally, we present an integrated model in Section 2.5.

### Chinese social relations

Individuals cannot live without social relationships. In China, a commonly used expression is ‘make use of guanxi’, which means people expect to establish and maintain relationships in anticipation that assistance can be obtained when the need arises. Hwang (1987) emphasizes that a large proportion of Chinese relationships is a combination of ‘instrumental’ relationships (with parties with whom one deals in order to achieve practical goals) and ‘affective’ relationships (with family members and close friends). Yu (1987) suggests that Chinese individuals strive to build relationships and share specific rules with others. The development of relationships can convert a person who was merely an acquaintance into a type of in-group member, and then exchanges of favors are used to maintain the relationship. Fei (1998) suggests that Chinese social relationships take the form of a differential pattern. In this pattern, each person forms his or her set of relationships and is simultaneously in the circle of other people’s relationships. Blood lineage, geography, economic level, political status, as well as intellectual and cultural level are the five major factors affecting the formation of the differential pattern. Among them, political status is the most influential.

China implements a system of multi-party cooperation and political consultation, which includes the Communist Party of China (CPC) and eight democratic parties, as well as people without party affiliation. The Communist Party of China is the founding and sole governing political party in China, and it is also the party with the largest number of members. A Chinese citizen who wants to join the Communist Party of China needs to go through a series of complicated processes. First, he needs to join the Chinese Communist Youth League and become a member of the Communist Youth League. Then, outstanding members of the Communist Youth League can be recommended to become active members. Active members have the opportunity to become probationary party members after inspection. Finally, prepare Party members can become full-fledged party members only after a one-year probation period. In China’s unique political system, political status is primarily related to people’s political affiliation. With the rapid development of social media, a series of activities and learning courses in the process of political profile conversion in China have gradually moved online and integrated with social media, and local party branches have started to actively use online learning applications to carry out political education activities, set quantitative assessment criteria such as learning points for applicants, and measure applicants’ political attitude through the information presented on social media platforms such as WeChat. If the number of hours of online learning or the number of learning points is not up to par, it may affect the individual’s evaluation results. The public’s political stance also affects social media expression and political participation behavior (Cao Yue, 2020). Given the many benefits of increased mindfulness, mindfulness has found its way into all spheres of society and even politics. In the United Kingdom, for example, mindfulness practice is widely spread among politicians, who see mindfulness as a good tool to help the entire polity and society (Bristow, 2019). In China, mindfulness has not yet been applied in the political field on a large scale, and there is also a lack of research on the correlation between mindfulness and political factions in academia.

### Mindfulness

Mindfulness, originally derived from the Buddhist tradition, is a meditation practice (Baer, 2003). It is generally defined as focusing attention in a non-judgmental and receptive way on the activities being in the moment and the inner thoughts and feelings of the individual (Bishop et al., 2004). The nature of mindfulness to be in the midst of things but to keep it at arm’s length in a non-judgmental manner gives mindfulness a number of benefits. For example, regular and continuous mindfulness practice is showed to reduce automatic responses in the emotional, physiological, and behavioral domains (Bartlett et al., 2021). Mindfulness can significantly reduce postpartum depression symptoms in mothers (Liu et al., 2022c). At the same time, mindfulness can help improve people’s subjective well-being (Liu et al., 2019). Many studies have demonstrated the positive effects of mindfulness on people in many fields, and this study wants to explore the specific effects of mindfulness on people in the social media environment.

To date, many tools for assessing mindfulness have been developed, such as the Mindfulness Attention Awareness Scale (MAAS; Brown and Ryan, 2003), the Toronto Mindfulness Scale (TMS; Lau et al., 2006), the Philadelphia Mindfulness Scale (PHLMS; Cardaciotto et al., 2008), the Kentucky Inventory of Mindfulness Skills (KIMS) developed by Baer et al. (2004), and their revised Five-Faced Mindfulness Questionnaire (FFMQ) based on KIMS (Baer et al., 2006). These scales differ mainly in the dimensions they measure, the population they are used for, and how and in which context they measure mindfulness. Among the many tools for assessing mindfulness, the MAAS has been considered by most scholars. The MAAS was initially validated with white college students and adults living in the United States. In addition, Deng et al. (2012) conducts a research test of the Chinese translation of the MAAS, demonstrating its effectiveness in assessing mindfulness in a population of Chinese college students. However, few scholars have explored the association between mindfulness and social media use in China, as well as the relationship between mindfulness and political context.

### Self-presentation on social media

The emergence of social media has led to the migration of offline social circles going online, with users finding a sense of identity and belonging to circles through more diverse and rich self-presentation. People tend to present an idealized self-image to others and make others form a specific impression of themselves through strategic self-presentation (Goffman, 1978). In real life, people rely on verbal communication, gestures, expressions, and dressing for face-to-face impression presentation and adjust their self-image through immediate observed audience reactions. The emergence of social media has turned this self-presentation into a strategic display characterized by reinforcing strengths and hiding weaknesses (Chenyu Dong, 2018).

WeChat is currently the most widely used social media platform in China. Displaying and sharing information through WeChat Moments has become a common method of social interaction. The WeChat Moments feature removes the time and space limitations of traditional interpersonal communication, which allows users to display themselves to a bigger audience without any in-person presence (Shan, 2019). In terms of communication content, dynamic visual displays such as short videos and GIFs make image building more vivid and comprehensive, adding more realism. Additionally, the contextual reproduction ability of the video can also better cope with contextual dissolution. In most contexts, individuals’ interpersonal relationships rely nowadays more on the online sphere than the offline sphere. Young people, who rely heavily on the internet, are the primary users of WeChat. They are sensitive to their self-image and the evaluation of others (Wang, 2017), and they are adept at purposeful and strategic self-presentation and impression management through this medium.

Extensive research over the years also demonstrates the many psychological benefits of mindfulness (Keng et al., 2011). Mindfulness is considered a potential regulator of self-presentation. Studies show that there is a negative correlation between mindfulness and loss of self-awareness dimensions, that is, high levels of mindfulness can help people better maintain self-awareness (Chen et al., 2019). Yang et al. (2017) explore how four dimensions of social media self-presentation affect college freshmen’s self-esteem and identity clarity by using a multi-faceted model of online self-presentation. Their research shows that mindful adults may be more effective in monitoring and regulating their online self-presentations. In particular, their research shows that those who are under pressure to be perfect and over-engage in extreme self-presentation can benefit greatly from increased mindfulness, which helps them shape better self-presentation (Flett et al., 2021). In addition, mindfulness plays an important role in improving negative outcomes associated with being left out on social media. Poon and Jiang (2020) research report states that when people do not get enough likes on social media, their negative emotions increase significantly, and mindfulness can moderate this effect.

Social media has become a social arena where self-expression and image construction are synchronized. Advanced technologies and mobile devices have created an instantly updated and closely connected online social world for users, increasing the accessibility of interpersonal communication and becoming the basis for modern people to build interpersonal relationships. Some people worry that socialization on social media platforms will gradually replace traditional face-to-face interpersonal communication, and people will use the symbolic system of new media to communicate, interact, and shape themselves.

### Social media pressure and social media addiction

Social networking platforms play an indispensable role in people’s daily life as a digital tool to execute social interactions (Pontes et al., 2018). With the popularity of social media, negative consequences such as social media addiction have attracted the attention of scholars. Social media addiction is a state in which an individual cannot control his or her social media use despite adverse effects on his or her life. The American Psychiatric Association defines social media addiction as a disorder requiring further research (American Psychiatric Association and Association, 2013). Specific symptoms of social media addiction include individuals spending more time than average on social media, having difficulty controlling their behavior, and a severe lack of offline socialization (Kraut et al., 1998; Iskender and Akin, 2010). In other words, for social addicts, social media becomes a compulsive activity (Kardefelt-Winther, 2014). This paper’s definition of social media addiction is based on behavioral addiction and considers it as a universal behavior.

Empirical studies show that users may discontinue to use platforms due to stress of social media use (Luqman et al., 2017). In recent years, there has been an increasing number of retreaters in WeChat Moments, and many people have gradually reduced the frequency of posting online and even blocked WeChat Moments. According to statistical reports on internet development in China, as of June 2020, the use of WeChat Moments has decreased by 2.3% compared to the end of 2017. It has also been found that social media users are likely to temporarily or permanently reduce their online social activities due to emotional torment (Oghuma et al., 2016; Swar et al., 2017). Previous research show that social media users are prone to tiredness after experiencing various technological, information, and social overloads (Bright et al., 2015). When users attempt to disengage from the adverse effects of social media, they often resort to switching online platforms or abandoning online social media in favor of offline activities, such as face-to-face interpersonal social behaviors, to distract themselves from the exhaustion and stress associated with social media use.

Mindfulness has shown to promote self-control (Holas and Jankowski, 2013). The research of Du et al. (2021) indicates that (1) individuals’ mindfulness intensity is positively correlated with social media self-control, (2) low mindfulness leads to social media self-control failure and further social addictive behavior, while (3) individuals with high mindfulness have better social media self-control. Compulsive use of social media creates greater pressure, and higher mindfulness can boost self-esteem, thereby reducing pressure-induced anxiety (Apaolaza et al., 2019). In addition, a study finds that mindfulness is negatively correlated with emotion-focused coping styles, that is, individuals with lower mindfulness tend to avoid problems rather than solve them (Sriwilai and Charoensukmongkol, 2016). Current research on avoidance behavior on social media is still insufficient. For example, Xiao and Mou (2019) study stops at users’ emotional changes. Therefore, this study sought to explore how mindfulness is related to social media pressure and social media addiction faced by people China’s unique political system. Meanwhile, based on the ‘stress-response-outcome’ framework, this study investigates how people adjust their social behavior online and offline after perceiving stress. According to the above literature, this study suggests the following hypotheses:

*H1:* In China's unique political and cultural context, there are differences in social media pressure faced by people with different political affiliations.

*H2:* In China's unique political and cultural context, there are differences in the level of social media addiction among people with different political affiliations.

*H3:* In China's unique political and cultural context, there are differences in mindfulness among people with different political affiliations.

*H4:* In the Chinese social environment, people's use of WeChat Moments can affect their online self-presentation behavior.

*H5:* In the Chinese social environment, people's level of mindfulness is linked to the use of WeChat Moments.

*H6:* In the Chinese social environment, people's level of mindfulness has an impact on their self-presentation.

*H7:* In the Chinese social environment, differences in people’s mindfulness can cause differences in their social media addiction behaviors.

*H8:* In the Chinese social environment, differences in people’s mindfulness can cause differences in perceived social media pressure.

*H9:* In the Chinese social environment, after people perceive stress, individuals with different mindfulness tend to choose different social behaviors to mitigate stress.

*H10:* In the Chinese social environment, after people experience social media addiction, people with different mindfulness tend to choose different social behaviors to deal with the addiction.

### An integrated model

Social psychology is known to be an important paradigm in communication research. With the evolution of social psychology, scholars have challenged the traditional stimulus–response (S-R) approach, which assumes only direct and automatic effects of media stimuli on people’s responses and have started acknowledging the important role of indirect effects or mediations. Likewise, communication researchers are increasingly interested in understanding the mechanisms through which people attend and respond to messages from various social media outlets. To investigate these mechanisms, our research explores how the orientation of individuals toward social networking sites (SNS) affects the way they process messages and, further, how this orientation affects cognitive and behavioral outcomes. In psychology, the S-R model has long been used to study changes in people’s attitudes. Woodworth and Schlosberg (1965) propose the S-O-R model based on the S-R model, which is the most widely used model.

The S-O-R model postulates that stimulation and human behavior (reaction, action) are linked by an organismic component. The model differs from the traditional S-R model mainly in two aspects: (1) It emphasizes that stimulation (S) does not directly respond to the behavior of individuals (R), (2) the behavior of individuals is based on consciousness as a mediator. The S-O-R model assume that the attitude of individuals is triggered by external sources of stimulation that directly or indirectly affect its physical and psychological states. When faced with various stimulations, individuals generate specific motivations and behaviors.

The factors influencing people’s perception of social media pressure and social media addiction are diverse and complex. Moreover, recent studies have shown that the relevant variables do not affect behavior in only one way, and different variables affect behavior in direct and indirect ways.

Our study refers to the S-O-R model: S is the social media use; O is self-presentation on social media, mindfulness, and social media pressure. That is how the attitudes and behavior of individuals (R) are formed, the variables are listed in Table 1.

**Table 1:** An S-O-R model.

Stimulus (S)	Orientation (O)	Response (R)
Social media use	Self-presentation	Social media addiction
	Mindfulness	Distraction within SNS and distraction outside SNS
	Social media pressure	

## Methodology

### Sampling and data collection

Despite studies on mindfulness, social media pressure, and social media addiction being conducted on various social media sites, there has been little investigation of WeChat, the most popular social networking site in China. WeChat has approximately 1.26 billion users worldwide (Statista, 2022) and is regarded as a typical instant messaging tool, similar to Facebook and WhatsApp. High levels of social media addiction may exist among WeChat users (Montag et al., 2018b).

For this paper, we conduct a survey among WeChat users. To investigate people’s SNS usage behavior, mindfulness, social media pressure, and social media addiction, we use a non-probability sampling method based on an online questionnaire. We collected our data in the period from 3 to 20 May 2022 through the online survey design and dissemination platform Wenjuanwang.^1^ This platform is used by many companies and universities in China to conduct online surveys (Mei and Brown, 2018). It provides a sampling pool of more than 260 million registered users in China, representing a diverse demographic background. Wenjuanwang randomly contacts users with brief information about the research and then records their responses to the survey (Vinnikova et al., 2020). We additionally incorporate a mixed-mode approach (e.g., using QQ and WeChat apps) to gather a broader sample of the relevant population. The first part of our questionnaire briefly introduces the research purpose and thanks the respondents for their participation. The second part comprises questions on the demographic characteristics of the respondents. The final part includes scale questions on the research constructs. Only the respondents who indicate to use a WeChat app on their smartphones at the time of filling out the questionnaire are included in our analysis. A total of 977 responses are included in our analysis. The sample proportions are male (*n* = 345) and female (*n* = 632). The majority of the participants are aged between 18 and 30 (72%, *n* = 703). As for education, most of the participants hold a bachelor’s degree (65.7%, *n* = 642; Table 2). The survey sample mainly focuses on college students, who are in a crucial period of political affiliation change.

**Table 2:** Demographic statistics of the respondents (*n* = 624).

	Variable	Number	Percentage (%)
Gender	Male	345	35.3
	Female	632	64.7
Age	Less than 18	24	2.5
	18–30	703	72.0
	31–40	163	16.7
	41–50	60	6.1
	Over 51	27	2.7
Education	High school and below	160	16.4
	Bachelor’s degree	642	65.7
	Master’s degree	154	15.8
	PhD	21	2.1

### Measures

The conceptualization and operationalization of the specific variables are defined as follows:

#### Media use

The study refers to the scale of PAN Shuya (2018) which measures the three aspects of habituation, information acquired, and entertainment. Participants respond on a five-level Likert scale (1 for strongly disagree and 5 for strongly agree) with the following questions: “I habitually visit my WeChat Moments every day”; “When I want to know what my friends and family are up to, I first look at their WeChat Moments”; “When I want to kill time or have fun, I browse my WeChat Moments.” The alpha coefficient of internal reliability is 0.763.

#### Mindfulness

We use the MAAS scale to measure the individual trait of mindfulness. Among the many instruments for measuring the trait of mindfulness, MAAS is the most widely used (Chen et al., 2021). The MAAS scale uses a structure of 15 items. Participants respond on a five-level Likert scale (1 for almost never and 5 for almost always). Lower scores represent greater mindfulness (Brown and Ryan, 2003). The alpha coefficient of internal reliability is 0.93 (see Table 3).

**Table 3:** MAAS.

Items	*M*	*SD*
I could be experiencing some emotion and not be conscious of it until sometime later.	3.34	1.143
I break or spill things because of carelessness, not paying attention, or thinking of something else.	2.99	1.242
I find it difficult to stay focused on what’s happening in the present.	3.44	1.157
I tend to walk quickly to get where I’m going without paying attention to what I experience along the way.	3.37	1.189
I tend not to notice feelings of physical tension or discomfort until they really grab my attention.	3.43	1.191
I forget a person’s name almost as soon as I’ve been told it for the first time.	3.37	1.215
It seems I am “running on automatic,” without much awareness of what I’m doing.	3.39	1.239
I rush through activities without being really attentive to them.	3.48	1.221
I get so focused on the goal I want to achieve that I lose touch with what I’m doing right now to get there.	3.43	1.081
I do jobs or tasks automatically, without being aware of what I’m doing.	3.17	1.180
I find myself listening to someone with one ear, doing something else at the same time.	3.30	1.173
I drive places on ‘automatic pilot’ and then wonder why I went there.	3.53	1.042
I find myself preoccupied with the future or the past.	3.67	1.033
I find myself doing things without paying attention.	3.45	1.096
I snack without being aware that I’m eating.	3.03	1.225

#### Self-presentation

In the WeChat Moments, self-presentation mainly refers to the naming of the user’s online identity, the choice of avatar, and emoji images. In this study, we measure self-presentation strategies according to the scale of Jiang (2013). The proactive strategy refers to the user’s initiative to show his or her good side and pay much attention to the comments of others. For example, “I often browse and update the status of WeChat Moments”; “I value my friends’ likes and comments in my WeChat moments”; “I pay attention to the personal image I present in my WeChat Moments”; “The photos I upload to my WeChat Moments are carefully selected”; “I actively interact with others on WeChat (Examle: give somebody a like or reply to messages)”; “When I post a message on my WeChat moments, I choose my words very carefully.” The alpha coefficient of internal reliability is 0.843.

#### Social media pressure

Social media pressure refer to factors that make users stressed. Perceived social overload is often considered as one of the stressors (Zhang et al., 2016). Based on the studies of Karr-Wisniewski and Lu as well as Zhang, perceived social overload is measured in two dimensions: information overload and functional overload(Karr-Wisniewski and Lu, 2010; Zhang et al., 2019).

Information overload refers to users’ exposure to excessive information on social media that they are unable to process. This study refers to the scale of Lin and Lu (2011) which considers two perspectives. The alpha coefficient of internal reliability is 0.829. Participants respond on a five-level Likert scale with questions such as: “I cannot browse through all the information in my WeChat Moments”; “Every time I open my WeChat Moments, there are too many updates in it”; “There is much information in my WeChat Moments, so it is hard for me to focus on the important information.”

Functional overload refers to the fact that the platform provides more features than users need. To be more specific: “It takes me a long time to understand and use the new features of WeChat (e.g., group payment, posting videos)”; “I do not have enough time to understand the new features of WeChat. (e.g., WeChat keyword search, hover window setting)”; “I feel that I do not know enough about all the features of WeChat to use it well”; “I often find WeChat features very complicated and troublesome to use.” The alpha coefficient of internal reliability is 0.886.

#### Social media addiction

Social media addiction refers to individuals who spend more time than average on social media and have difficulty controlling their behavior. Participants respond on a five-level Likert scale to the following questions: “When browsing my WeChat Moments, I often neglect important things”; “Spending much time browsing WeChat Moments has a positive effect on my real-life social behavior”; “When I am studying or working, I often get distracted by browsing my WeChat Moments”; “When I do not browse my WeChat Moments, I feel uneasy”; “I have tried to reduce the frequency of browsing WeChat Moments, but I failed”; “Browsing my WeChat Moments affects my offline private life.” (Turel and Serenko, 2012). The alpha coefficient of internal reliability is 0.904.

#### Distraction within SNS and distraction outside SNS

Individuals tend to cope with stress through adaptive behaviors (Lazarus and Folkman, 1984). Attentional distraction behavior is one of these adaptive behaviors. Research shows that people tend to be distracted both inside and outside of social media platforms when they are social media fatigued. In-platform distraction refers to when people get bored with one social media, they tend to switch to another social media, while off-platform distraction refers to when people get bored with one social media, they tend to switch to another social media. Switching to other platforms than social media platforms (e.g., offline social platforms). This study refers to the scale of Salisbury et al. (2002), which measures both in-platform and out-of-platform aspects. We ask participants to recall their coping practices and thoughts when they were unhappy about viewing information from their WeChat Moments in the past. Participants respond on a five-level Likert scale to the following questions: “I stopped browsing my WeChat Moments and went to the content of the official WeChat accounts that I follow”; “I stopped browsing my WeChat Moments and chatted with my family or friends instead”; “I stopped browsing my WeChat Moments and did other things (e.g., walking, reading books, watching TV)”; “I stopped browsing my WeChat Moments and used other social media platforms (e.g., Sina Weibo, QQ)”; “I stopped browsing my WeChat Moments and used video and audio applications other than WeChat (e.g., TikTok, video sites such as IQIYI and Tencent).” The alpha coefficient of internal reliability is 0.833.

## Data analysis and results

### Differences in mindfulness, social media pressure, and social media addiction across different political affiliations

Based on individual demographics, there are significant differences in social media use, social media pressure, and social media addiction. This study considers the unique political and cultural situation in China.

The mean and standard deviation of all scales are listed in Table 4. A univariate ANOVA reveals significant differences in mindfulness, social media pressure, and social media addiction depending on political affiliation (see Table 5 below), supporting H1, H2, and H3. First, the mindfulness of members of the CPC, probationary CPC members, and members of the Communist Youth League of China (CYLC) is higher than that of members of the democratic party. Moreover, a comparison reveals that the mindfulness of probationary CPC members is higher than that of the masses (*F* = 5.93, *p* < 0.001). Second, the social media addiction status of members of the democratic party and the masses is higher than that of probationary CPC members and members of the CYLC (*F* = 8.1, *p* < 0.001). Finally, our study finds that members of the democratic party and the masses perceive more social pressure on social media than probationary CPC members and members of the CYLC (*F* = 9.04, *p* < 0.001).

**Table 4:** Descriptive of the sample.

Scale	Question	*M*	*SD*
Media use	I habitually visit my WeChat Moments every day	3.95	1.19
	When I want to know what my friends and family are up to, I first look at their WeChat Moments	3.8	1.16
	When I want to kill time or have fun, I browse my WeChat Moments	3.7	1.18
Self-presentation	I often browse and update the status of WeChat Moments	2.72	1.37
	I value my friends’ likes and comments in my WeChat moments	3.45	1.25
	I pay attention to the personal image I present in my WeChat Moments	3.73	1.14
	The photos I upload to my WeChat Moments are carefully selected	3.9	1.14
	I actively interact with others on WeChat (Examle: give somebody a like or reply to messages)	3.45	1.2
	When I post a message on my WeChat moments, I choose my words very carefully	3.58	1.16
Social media pressure	I cannot browse through all the information in my WeChat Moments	3.23	1.33
	Every time I open my WeChat Moments, there are too many updates in it	3.18	1.28
	There is much information in my WeChat Moments, so it is hard for me to focus on the important information	3.05	1.31
	It takes me a long time to understand and use the new features of WeChat (e.g., group payment, posting videos)	2.73	1.34
	I do not have enough time to understand the new features of WeChat. (e.g., WeChat keyword search, hover window setting)	3	1.32
	I feel that I do not know enough about all the features of WeChat to use it well	2.91	1.28
	I often find WeChat features very complicated and troublesome to use	3.22	1.22
Social media Addiction	When browsing my WeChat Moments, I often neglect important things	2.73	1.33
	Spending much time browsing WeChat Moments has a positive effect on my real-life social behavior	2.96	1.21
	When I am studying or working, I often get distracted by browsing my WeChat Moments	2.97	1.33
	When I do not browse my WeChat Moments, I feel uneasy	2.6	1.35
	I have tried to reduce the frequency of browsing WeChat Moments, but I failed	2.84	1.35
	Browsing my WeChat Moments affects my offline private life	2.78	1.32
Distraction within SNS and Distraction outside SNS	I stopped browsing my WeChat Moments and went to the content of the official WeChat accounts that I follow	2.92	1.31
	I stopped browsing my WeChat Moments and chatted with my family or friends instead	3.33	1.26
	I stopped browsing my WeChat Moments and did other things (e.g., walking, reading books, watching TV)	3.81	1.12
	I stopped browsing my WeChat Moments and used other social media platforms (e.g., Sina Weibo, QQ)	3.6	1.22
	I stopped browsing my WeChat Moments and used video and audio applications other than WeChat (e.g., TikTok, video sites such as IQIYI and Tencent)	3.64	1.22

**Table 5:** Univariate ANOVA results.

Variable	Groups	Mean	*S.D.*	*F*-test	
Mindfulness	Members of the CPC	3.14	1.18	5.93***	Members of the CPC, Probationary CPC members, Members of the CYLC>Members of the Democratic Party; Probationary CPC members> The masses
	Probationary CPC members	2.83	0.90		
	Members of the CYLC	3.17	0.78		
	Members of the Democratic Party	4.01	0.97		
	Persons without party affiliation	3.22	0.82		
	The masses	3.57	1.02		
Social media addiction	Members of the CPC	3.05	1.20	8.10***	Members of the Democratic Party, The masses>Probationary CPC members, Members of the CYLC
	Probationary CPC members	2.49	0.94		
	Members of the CYLC	2.84	1.06		
	Members of the Democratic Party	3.99	0.95		
	Persons without party affiliation	2.58	1.18		
	The masses	3.47	1.11		
Social media pressure	Members of the CPC	3.03	1.24	9.04***	Members of the Democratic Party, The masses>Probationary CPC members, Members of the CYLC
	Probationary CPC members	2.32	0.92		
	Members of the CYLC	2.79	1.03		
	Members of the Democratic Party	3.96	0.90		
	Persons without party affiliation	3.01	0.96		
	The masses	3.44	1.13		

**p<0.01 and ***p<0.001.

### Data model testing and analysis of results

Due to the multifaceted nature of the factors affecting social media pressure and social media addiction, this study uses structural equation modelling (SEM) for examination. The SEM in this study consists of two parts: (1) the measurement model describes the relationship between latent variables and indicators, and (2) the structural model describes the path relationships between different latent variables (Wu, 2009).

After performing SEM in AMOS 21, an important issue is the criterion for accepting or rejecting a model. Wu states that a model should satisfy the following conditions: (1) the chi-square value divided by the model degrees of freedom (*χ*^2^/df) should be below 5 and preferably below 3; (2) root mean square error of approximation (RMSEA) values should be lower than 0.08 and preferably below 0.06; (3) standardized root mean squared residual (SRMR) values below 0.10 are considered to be favorable; and (4) Tacker–Lewis index (TLI) and comparative fit index (CFI) values above 0.90 and preferably above 0.95 indicate a good model fit (Wu, 2009).

**Figure 1:**
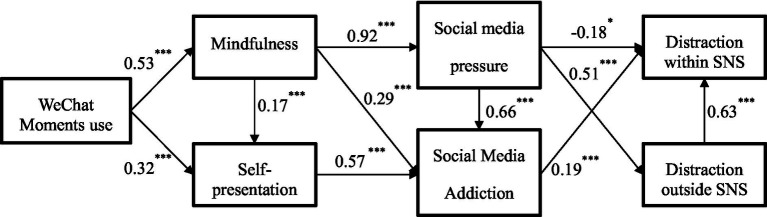
Relationships between variables. Correlations appear below the diagonal; the square roots of AVE values appear on the diagonal and are presented in bold font. *** *p* < 0.001.

This study’s latent variables include WeChat Moments use, mindfulness, self-presentation, social media pressure, social media addiction, as well as distraction within SNS and distraction outside SNS. The study adopts a fixed loading method (unit loading identification constraint), and the model fit results are ideal (*p* = 0.000, df = 923, *χ*^2^ = 2777.962; *χ*^2^/df = 3.01, TLI = 0.919, CFI = 0.928, GFI = 0.877, AGFI = 0.856, RMSEA = 0.045). The relationships between the variables are shown in Figure 1. From the results, we conclude that the overall fit of the CFA model is acceptable. Furthermore, we test the discriminant validity. In Table 6, all correlations between each pair of constructs are less than the square roots of the AVE values, which supports the discriminant validity.

**Table 6:** Means, standard deviation, and correlations.

	Mean	*SD*	1	2	3	4	5	6
1. WeChat Moments use	3.81	0.97	**0.679**					
2. Mindfulness	3.36	0.83	0.472***	**0.757**				
3. Self-presentation	3.47	0.91	0.633***	0.666**	**0.682**			
4. Social media Addiction	2.82	1.08	0.439***	0.700**	0.663**	**0.718**		
5. Social media Pressure	3.03	0.98	0.250***	0.752**	0.520**	0.685**	**0.658**	
6. Distraction within SNS and distraction outside SNS	3.68	0.86	0.320***	0.433**	0.410**	0.373**	0.357**	**0.554**

**p<0.01 and ***p<0.001.

We derive five insights from the data results. (1) People’s use of WeChat Moments generates significant positive correlations for the mindfulness and self-presentation variables. Browsing WeChat Moments is positively correlated with positive self-presentation in WeChat Moments (*β* = 0.32, *p* < 0.001), and negatively related to perception of mindfulness (*β* = 0.53, *p* < 0.001), which supports H4 and H5. (2) While browsing WeChat Moments, people’s mindfulness levels were negatively correlated with social media addiction (*β* = 0.29, *p* < 0.001), and positively correlated with positive self-presentation on WeChat Moments (*β* = 0.17, *p* < 0.001). Thus, H6 and H7 are accepted. (3) After being exposed to WeChat Moments information, People’s mindfulness levels are negatively correlated with stress problems (*β* = 0.92, *p* < 0.001), which supports H8. (4) The pressure people feel on social media is positively correlated with the willingness to switch to offline interpersonal interactions (*β* = 0.51, *p* < 0.001). However, people’s offline interaction frequency is positively correlated with their willingness to return to online social media (*β* = 0.63, *p* < 0.001). In addition, the pressure people feel is positively correlated with social media addiction (*β* = 0.66, *p* < 0.001), which supports H9. (5) Finally, people’s perceived level of self-social media addiction is positively correlated with willingness to switch social media platforms such as Sina Weibo, Tik Tok, and QQ (*β* = 0.19, *p* < 0.001), which supports H10.

## Discussion

Based on the results of the data analysis, this study presents the following discussion and research findings.

### Differences mindfulness between social media use and different political affiliations

Our results show that WeChat Moments use weakens public mindfulness. The reason may be that browsing WeChat Moments distracts users from their work or study. By preventing users from focusing their awareness and attention on their current tasks, this can have a negative impact on mindfulness. Existing research also supports this conclusion that regular use of social media leads to a decrease in mindfulness (Du et al., 2021).

The results also show that there are differences in mindfulness among people from different political backgrounds. Among them, the mindfulness of CPC members is higher than that of people from other political backgrounds. The reason may be that the CPC has certain restrictions on the use of social media for its members. Social media is no longer just a tool for interpersonal communication in China, but it has become a platform for theoretical learning and ideological education. For example, Xuexi Qiangguo is a theoretical learning platform based on the points system (Liu, 2019). Party organizations at all levels of the CPC use this platform to quantify the theoretical learning level of party members. Local party organizations formulate score standards and require their party members to achieve these goals. Therefore, in order to join the CPC, applicants have to spend a lot of energy on improving the points on Xuexi Qiangguo, while formal party members continue to improve their points on Xuexi Qiangguo (Chen, 2021). In addition, CPC members are often asked to repost various CPC Central Committee meeting pushes and announcements on their WeChat Moments, resulting in their Moments being filled with work-related content rather than personal photos and entertainment news. Combined with the above conclusions, lower use of WeChat Moments also contributed to higher levels of mindfulness.

### Self-presentation with social media use

In the WeChat Moments, the primary content of self-presentation is expressed in the naming of the users’ online identities. In addition, the choice of avatar is also related to the construction of self-images. Embellished real-life photos, pictures presenting personality, and even emoticons expressing emotions become the mapping of self-image in daily life (Du, 2020). After successfully establishing identities and adding friends, users perform self-presentation and maintain interpersonal relations by posting content in their WeChat Moments, reposting media articles, browsing others’ WeChat Moments, and liking and commenting on interactions. In this process, communicators strategically choose how to present themselves and set the visibility of their messages (Treem et al., 2020).

Users use WeChat Moments to show their identity and present their self-image, expressing inner feelings explicitly or implicitly, directly or abstractly. In another way, this shapes the image in the minds of others through the symbols that reflect users’ styles and attitudes to enhance identification with their identity.

Our results show that the more mindful people are, the less inclined they are to present themselves in their WeChat Moments. This may be due to the fact that people with higher mindfulness are not overly concerned with what others think of themselves and do not need excessive self-presentation to seek attention or gain approval from others. Carson and Langer (2006) research shows that when people are mindful, they are less inclined to manipulate their own image or maintain fragile self-esteem in order to gain positive evaluations from others.

### Social media pressure and social media addiction associated with social media use

This study finds that there is a positive correlation between excessive social media use and the audience’s perceived stress. In addition, social media addiction behavior increases when the public feels more social media pressure. This finding supports some research that compulsive social media use leads to fatigue with social media (Bai, 2020). Zhang et al. (2019) identify perceived social overload as one of the sources of stress that cause social media fatigue and state that social media addiction can be classified explicitly into information overload, functional overload, and social overload. The specific differences are as follows: (1) information overload is a state in which users are exposed to too much information that they cannot process effectively (Soto-Acosta et al., 2014). Research indicates that due to the limited cognitive abilities of individuals, users are negatively affected when they perceive too much irrelevant or unnecessary information (Maier et al., 2015); (2) functional overload refers to platforms providing more functionality than users need (Thompson et al., 2005). When the level of change in the users’ perception of social network functional requirements and the speed of version changes are too fast, users feel social media pressure, because they need to spend a lot of time readjusting; (3) social overload indicates that the user’s social network expansion forces the user to spend more time and energy meeting social needs, providing social support, and maintaining social relationships. Social overload can be further categorized into communication overload (refers to individuals receiving communication demands on social media that exceed their processing capabilities) and self-presentation (refers to individuals’ efforts to build, change, or maintain an image in the minds of others) according to the subject’s affiliation (Cho et al., 2011). Wang and Stefanone (2013) find that users with more positive self-presentation login to Facebook more frequently. Zhang et al. (2019) suggests that self-presentation can promote social overload.

### Mindfulness for social media addiction

As technology develops exponentially, users can access an infinite amount of information at the click of a button. With this privilege comes the potential cost of information overload, social media addiction, and increased distraction. Social media addiction is becoming an increasing problem.

One cure is mindfulness meditation, a training that is being proven by science to be a powerful tool for enhanced well-being and mental focus. However, loving-kindness meditation based on mindfulness meditation does not significantly improve the level of mindfulness (Chen et al., 2021). But loving-kindness meditation can significantly improve people’s sense of social presence (Liu et al., 2022d). Mindfulness is a training that helps users to become more present, self-aware, and better able to respond rather than react to everyday situations. Experts, such as the American addiction psychiatrist Judson Brewer, show that mindfulness can improve impulse control and reduce addictions ranging from social media to drugs (Brewer, 2019). Mindfulness is a practice that supports people to act quickly in order to break bad habits. Mindfulness practice tends to reduce anxiety levels and stress (Liu et al., 2022e). This result also supports our research that strengthening mindfulness can effectively reduce social media addiction.

### Social avoidance behavior

In our study, we find that when users receive negative feedback, e.g., opposing comments on their social media posts, they are more likely to develop negative emotions. Users are then more inclined to change their self-presentation behavior, such as no longer following their WeChat Moments (ignoring behavior), blocking individuals (blocking behavior), reducing their voice (diving behavior), or even closing their WeChat Moments (withdrawal behavior).

Although users tend to stop using online media and turn to offline communication when they feel more social media pressure, they do not permanently stop socializing online. Instead, they even revert to social media and become addicted to it. After the perceived stress is relieved, users return to social media and continue to use it or engage in other social media platforms to overcome the perceived social exhaustion on the previous platform. Previous research on Facebook suggests that passive use of Facebook leads to a decline in emotional well-being over time, consequently causing withdrawal behavior. This finding is sufficiently supported by existing empirical research (Sagioglou and Greitemeyer, 2014; Gil-Or et al., 2015; Dhir et al., 2018).

### Research limitations and future research

A few limitations of the present study require consideration. First, as the current study is conducted with a sample of college students in China, it remains to be determined whether our findings can be generalized to different age groups. Results may differ in heterogeneous groups with different population or cultural backgrounds. Second, our results rely upon self-report data, which might include inaccurate responses from participants. Third, we cannot prove any causal relationships, e.g., between social media pressure factors and social media addiction factors, because this study has a cross-sectional design. Even a reverse directionality is possible (Montag et al., 2018a). Therefore, the explanation proposed here should be treated with appropriate caution and future research should focus on longitudinal investigations.

## Conclusion

In summary, the present study examined a multiple mediation model through which mindfulness is associated with social media pressure and social media addiction. Our results revealed that a high social media use increases social media pressure and potentially causes social media addiction. Mindfulness can effectively relieve people’s social media pressure and therefore can serve as a lever to alleviate social addiction.

## Data availability statement

The raw data supporting the conclusions of this article will be made available by the authors, without undue reservation.

## Ethics statement

Ethical review and approval was not required for the study on human participants in accordance with the local legislation and institutional requirements. Written informed consent from the patients/participants or patients/participants legal guardian/next of kin was not required to participate in this study in accordance with the national legislation and the institutional requirements.

## Author contributions

CY led the work carried out on the study, including its conceptualization, research design, data collection and data analysis, and took the lead in writing the manuscript. YL was involved in the research design, manuscript writing and proofreading, contributed to its write-up.

## Funding

This study was financially supported by the National Social Science Fund of China (Award #21CXW004).

## Conflict of interest

The authors declare that the research was conducted in the absence of any commercial or financial relationships that could be construed as a potential conflict of interest.

## Publisher’s note

All claims expressed in this article are solely those of the authors and do not necessarily represent those of their affiliated organizations, or those of the publisher, the editors and the reviewers. Any product that may be evaluated in this article, or claim that may be made by its manufacturer, is not guaranteed or endorsed by the publisher.

## Footnotes

^1^ https://www.wenjuan.com/
